# Assessment of subjective and objective masticatory function among elderly individuals with mild cognitive impairment

**DOI:** 10.1007/s40520-022-02290-x

**Published:** 2022-11-11

**Authors:** Nan-Ju Lee, Hyo-Jung Kim, Yiseul Choi, Taek-Bin Kim, Bock-Young Jung

**Affiliations:** 1 Department of Advanced General Dentistry, Human Identification Research Institute, Yonsei University College of Dentistry, 50 Yonseiro, Seodaemun-Gu, Seoul, 03722 South Korea; 2 Department of Preventive Dentistry and Public Oral Health, Yonsei University College of Dentistry, Seoul, South Korea; 3 275 dental clinics, Gyeonggi-Do, South Korea

**Keywords:** Masticatory function, Mild cognitive impairment (MCI), Masticatory ability index (MAI), KMMSE

## Abstract

**Background:**

Masticatory function is known to be related to cognitive ability; therefore, factors for improving masticatory function should be identified.

**Aims:**

This study aimed to identify factors influencing masticatory function associated with mild cognitive impairment (MCI) in elderly individuals.

**Methods:**

A total of 123 elderly participants [mean age: 76.5 ± 6.5 years; 82 females (66.7%), 41 males (33.3%)] were included. Cognitive function was evaluated by the Korean version of the Mini-Mental State Examination (KMMSE). Questionnaires for subjective evaluation were administered, and dynamic objective masticatory function evaluations, including chewing tests and bite force measurements, were performed. Intergroup differences were evaluated by the Wilcoxon rank-sum and chi-square test, and correlations between cognitive ability and masticatory function were evaluated by multilinear logistic regression.

**Results:**

The number of teeth, number of posterior teeth, bite force, masticatory ability index (MAI) and posterior support status showed significant differences between the normal (KMMSE > 23) and MCI (KMMSE ≤ 23) groups. However, only the MAI, representing dynamic masticatory performance, was significantly associated with MCI regardless of age, sex and removable prostheses. The number of teeth and posterior teeth, bite force, subjective masticatory ability and posterior occlusal support showed no significant association with MCI.

**Discussion:**

These results suggested the importance of chewing function for preventing the progression of cognitive impairment.

**Conclusions:**

Considering that only the MAI was significantly associated with MCI, it is more important to improve chewing efficiency by harmonizing therapeutic prosthetics with the surrounding masticatory system than simply increasing the number of teeth to prevent or delay cognitive impairment in elderly individuals.

**Supplementary Information:**

The online version contains supplementary material available at 10.1007/s40520-022-02290-x.

## Introduction

As the aging population is growing rapidly worldwide, aging-related health problems such as cognitive impairment and dementia have begun to stand out as social issues in terms of welfare.

Mild cognitive impairment (MCI) is the stage between the expected cognitive decline of normal aging and the more serious decline of dementia. MCI should be clinically noted because it is likely to progress to dementia and thus may provide insight into the pathogenesis of dementia as a predementia stage. Previous long-term follow-up studies have shown that the rate of progression to dementia ranges from 10 to 80% among MCI patients [1, 2]. Notably, MCI can often revert to normal cognitive function, and the estimated rate of MCI improvement or reversion has been reported to range from 4.5% to 53%, depending on various factors [3, 4]. These suggested risk factors and recovery factors for cognitive impairment include sociodemographic (age, sex, education level), genetic (APOE ε4 allele), lifestyle (smoking, alcohol, diet, exercise), physical and mental (cardiovascular disease, hypertension, diabetes mellitus, Mini-Mental State Examination [MMSE] score) factors that are intricately related to each other and form a complex model [2–5].

Recently, many studies have reported that masticatory function is mutually associated with cognitive ability, and although which comes first is not yet known, masticatory dysfunction is one of the risk factors for cognitive decline in elderly individuals [6–9].

Masticatory dysfunction refers to the decrease in or deterioration of masticatory function caused by a structural factor (e.g., number of remaining teeth, posterior occlusal contact), a functional factor (e.g., masticatory performance, bite force) or both combined. Meanwhile, subjective factors, such as the mental or psychological condition of an individual, could influence masticatory function [10–12].

The number of remaining teeth has been suggested as a significant independent risk factor for cognitive impairment by explaining two possible mechanisms, i.e., neuroinflammation caused by increased proinflammatory mediator levels and malnutrition due to tooth loss [8, 13, 14]. In addition, a poor chewing ability and low occlusal force are significantly associated with cognitive function [10, 15, 16], and oral rehabilitation using prostheses, such as removable prostheses (RPs) or dental implants, is effective in preserving cognitive function [17].

Several mechanisms underlying this relationship between mastication and cognitive function have been investigated. Cognitive impairment is due to neurophysiological changes in the brain, including cortical atrophy and the deterioration of neurons and synapses in brain regions controlling learning, memory and emotional behavior, which are also known to be related to chewing function [18, 19]. In addition, functional movement during chewing increases regional cerebral blood flow levels [20] and changes the blood flow in the internal carotid artery, stimulating oxygenation and perfusion of the brain area related to memory, especially the hippocampus [21, 22]. Although the underlying mechanism activated by mastication is still unclear, the fact that mastication activates brain function has reached a broad consensus.

Cognitive function has been assessed using various methods, including a neuropsychological test battery for extensive assessment [23] and the MMSE [23–25] and Montreal Cognitive Assessment [26] (MoCA) for general assessment. The MMSE is a standardized, widely used tool for the evaluation of general cognitive function in terms of orientation, attention, memory, language and visual-spatial skills because of its reliability in predicting cognitive impairment and the advantages of quantitatively evaluating the degree of cognitive impairment in 5–10 min [25]**.** These tests have been modified into different versions, for example, the Korean version of the Mini-Mental State Examination (KMMSE) [27] and the Japanese version of the MoCA (MoCA-J), to fit the customs of each population [16].

Clinically, the identification of factors associated with MCI progression to dementia and MCI reversion to normal cognitive function has important implications. There have been many studies on the association between oral health and cognitive status, but most of these studies have examined single-element relationships and have shown conflicting results. Variations in methodology, especially diversity in research populations, are considered the main reason for the lack of causality and inconsistent outcomes. Additionally, there are no standard data regarding oral function from a normal healthy population comprising various age groups to enable a quantitative comparison. This study aimed to identify the significantly influential variables among various factors of subjective and objective masticatory function that are potentially associated with MCI and to suggest an oral rehabilitation guide for the reversion of MCI in elderly individuals.

## Materials and methods

### Study participants

This cross-sectional study was performed according to the guidelines of the Declaration of Helsinki and was approved by the institutional review board committee of the Yonsei University College of Dentistry (No. 2-2019-0009). Among the patients over 65 years of age who visited the Department of Advanced General Dentistry of the Yonsei University Dental Hospital from March 2019 to February 2020, 123 participants who met the following criteria were included: (1) able to communicate, answer the questions and fill out the questionnaires on their own; and (2) had completed prosthetic treatment at least 4 weeks prior and had no chewing problems.

To avoid bias from factors not related to masticatory function, the exclusion criteria were as follows: (1) history of a congenital or acquired disease, such as cerebral infarction, or psychiatric illness, including depression and dementia, that could make it difficult for the participant to communicate with researchers; (2) difficulty in performing dynamic masticatory function tests with maximal effort due to health problems, such as cardiovascular disease, general weakness after surgery and Parkinson disease [24]; and (3) temporomandibular joint pain and tooth mobility of grade 2–3. Written consent was received directly from each participant. The sample size was calculated using G*power 3.1 software (Kiel University, Kiel, Germany) with α set to 0.05, the power set to 0.99 and the effect size set to 1.68.

### Study design

All participants underwent cognitive and masticatory function assessments. Cognitive function was assessed by 1 trained researcher using the KMMSE. For the masticatory function evaluation, both subjective and objective assessments were performed. The subjective masticatory ability assessment was conducted using a simple questionnaire, the Key Food Intake Ability (KFIA) questionnaire, to determine the participant’s own masticatory ability. A chewing test and bite force measurement were performed to assess the dynamic objective masticatory function, and the number of remaining teeth, number of posterior teeth, posterior occlusal contact and presence or absence of RPs were recorded for the static objective masticatory assessment.

### Evaluation of cognitive function

The KMMSE consisted of 30 questions in the following 6 domains: registration; attention and calculation; recall; language; ability to follow simple commands; and orientation. Each question was rated as 0 or 1 point, and the KMMSE score was recorded as the total number of points, ranging from 0 to 30, with lower scores indicating greater cognitive impairment. The MCI group included participants with a cutoff score of 23 based on a previous study [27]. Those who belonged to the MCI group in this study were recommended to visit a neurologist for further evaluation.

### Dental status assessment

The number of remaining teeth was determined by counting the teeth that were natural or restored teeth except for third molars and root rests. Posterior occlusal supports were recorded using the Eichner index based on the condition of posterior occlusal contact between the maxilla and the mandible, with the following 3 classifications: Eichner A, occluding pairs in four bilateral posterior supports; Eichner B, one to three occluding pairs or occluding contacts in the anterior region; and Eicher C, no occluding pairs [28].

### Mixing ability test

**Fig. 1:**
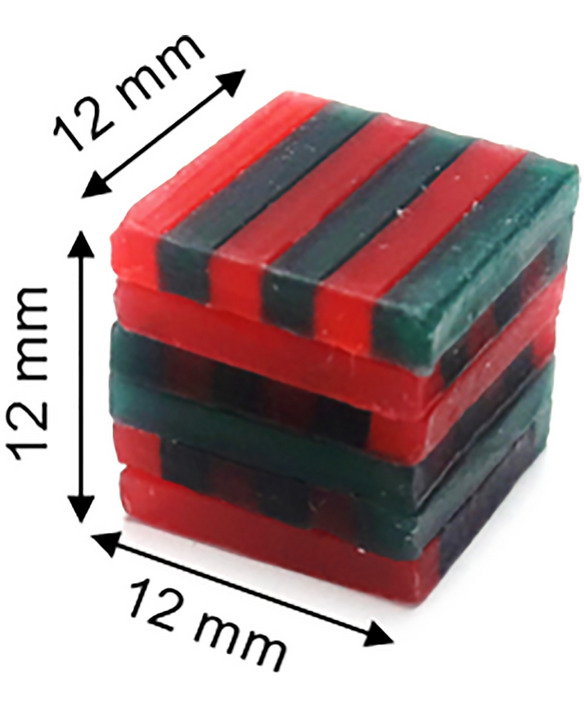
Wax specimen

**Fig. 2:**
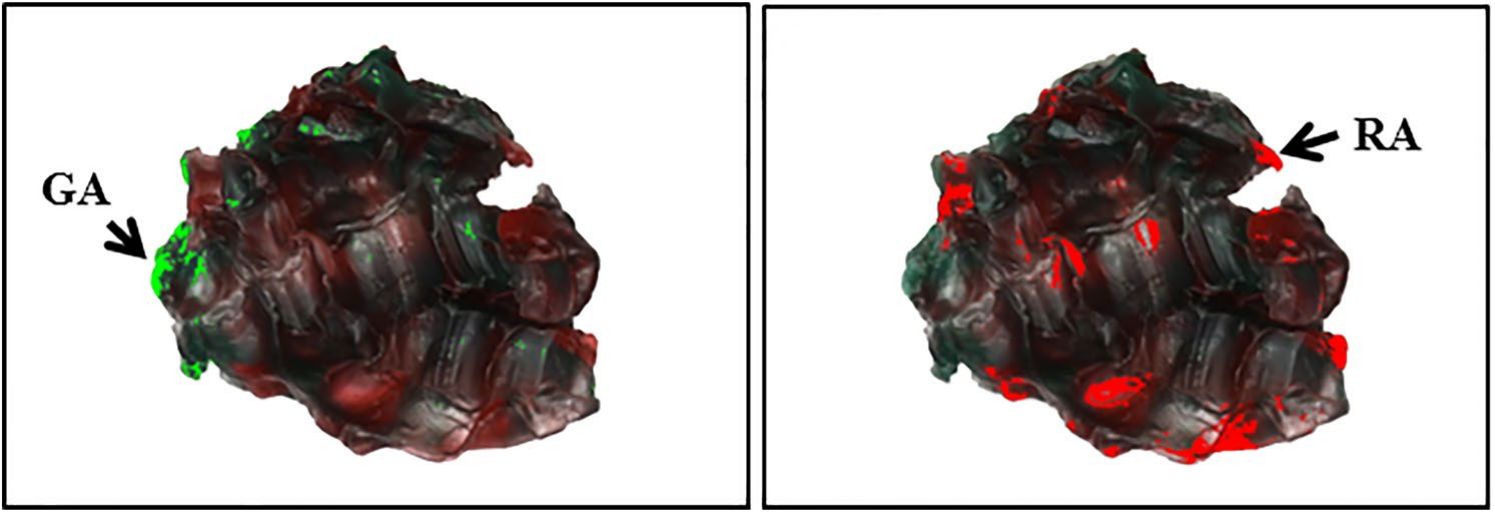
Identification of areas without color mixing in color images. Example of color image analysis using a digital image analyzer (Image-Pro Plus® version 6.0, Media Cybernetics, Inc., Bethesda, MD, USA). The unmixed areas were identified and marked with GA (green area) and RA (red area). The unselected areas were judged to be mixed (i.e., as indicated by a combination of the two colors of wax)

The mixing ability test developed by Sato et al. [29] was used to measure chewing ability and dynamic masticatory performance. The mixing ability index (MAI) was calculated by analyzing the degree of color mixing and the shape and width of a chewed wax specimen, a two-color wax cube (12*12*12 mm^3^) (Fig. 1). Each participant was instructed to chew the wax specimen ten times with a normal chewing pattern using one’s own habitual masticatory side with the head upright in an unsupported natural position. This chewing test was repeated twice, and the chewed wax specimens were kept in a refrigerator and analyzed within three days to avoid any deformation. Images of both the front and back sides of the chewed wax specimen without shadows were captured using a digital single-lens reflex camera (D80, Nikon Co., Tokyo, Japan) and saved as JPEG files [30]. Both monochrome images and color images of each specimen were obtained using a digital image analyzer (Image-Pro Plus® version 6.0, Media Cybernetics, Inc., Bethesda, MD, USA) (Fig. 2). Areas without color mixing, i.e., green or red areas, were selected in color images using an eyedropper tool built in the analyzer and calculated by a single independent examiner to eliminate measurement error. The MAI was calculated using a scale of 0–100 points, and the average of two specimens was obtained. The MAI assessment is a relative comparison, with a higher MAI indicating better masticatory performance [30].

### Bite force measurement

Participants were instructed to bite with their own maximum force several times at the maximal intercuspal position for 3 s while seated in a comfortable position with the head upright, keeping Frankfort’s horizontal plane parallel to the ground. An adequately sized pressure-sensitive film (Dental Prescale 50H, GC, Japan) was positioned in the mouth, and the bite force was measured and analyzed using a bite force analyzer (OCCLUSER 709, GC, Japan) [31].

### Key food intake ability (KFIA)

Subjective masticatory ability was assessed using the self-assessed questionnaire asking if the participant had any difficulties chewing five key foods, including peanuts, carrots, caramel, dried squid, and diced radish Kimchi [32], and the KFIA score was recorded using a five-point Likert scale depending on the degree of discomfort. The average score for the five key foods was recorded as the KFIA score, with a higher score indicating better subjective masticatory function (Table 1).

**Table 1:** Questionnaire for the evaluation of the KFIA score

Food list	Cannot chew at all (1)	Difficult to chew (2)	Cannot say either (3)	Can chew some (4)	Can chew well (5)
1 Peanut					
2 Carrot					
3 Dried squid					
4 Caramel					
5 Radish kimchi					
Total score			Average		

### Statistical analysis

All statistical analyses were carried out using SAS 9.4 software (SAS Institute, Inc., Cary, NC, USA) with a significance level of 0.05. The Wilcoxon rank-sum test was used to determine differences between the groups in terms of continuous dependent variables, and the chi-square test was used for categorical variables. To determine the statistical correlation between cognitive ability and masticatory function, multiple generalized linear and logistic regression analyses were performed. The dependent variables were age, the KFIA score, number of teeth, number of posterior teeth, bite force, MAI and Eichner index, and the independent variable was the KMMSE score.

## Results

### Characteristics of participants according to the KMMSE score (Table 2)

**Table 2:** Characteristics of participants according to the KMMSE score (*n* = 123)

Dependent variable	Total (*n* = 123)	Normal cognition group (*n* = 92)	MCI group (*n* = 31)	*P* value*
Sex M: F, *N* (%)	41 (33.33): 82 (66.67)	32 (34.78): 60 (65.22)	9 (29.03): 22 (70.97)	0.5569†
Age	76.54 ± 6.53, 76.00 (11.00)	75.51 ± 5.59, 75.50 (8.00)	79.61 ± 8.08, 82.00 (17.00)	0.0108
KMMSE score	25.91 ± 4.06, 27.00 (6.00)	27.88 ± 1.47, 28.00 (2.00)	20.06 ± 3.65, 21.00 (5.00)	< .0001
KFIA score	3.53 ± 1.02, 3.60 (1.40)	3.60 ± 1.07, 3.60 (1.40)	3.33 ± 0.81, 3.20 (1.00)	0.0764
Remaining teeth	19.73 ± 7.88, 22.00 (10.00)	20.58 ± 7.59, 22.50 (9.00)	17.23 ± 8.30, 19.00 (12.00)	0.0296
Posterior teeth	10.29 ± 4.88, 12.00 (6.00)	10.87 ± 4.87, 12.00 (6.50)	8.58 ± 4.57, 9.00 (7.00)	0.0097
Bite force	644.58 ± 387.25, 560.80 (503.50)	683.22 ± 389.95, 600.55 (517.2)	529.90 ± 361.16, 509.00 (462.10)	0.0479
MAI	66.70 ± 9.68, 68.43 (11.89)	70.10 ± 7.41, 71.10 (8.87)	56.60 ± 8.59, 58.40 (11.94)	< .0001
Eichner A: B + C, *N* (%)	67 (54.47):56 (45.53)	57 (61.96):35 (38.04)	10 (32.26):21 (67.74)	0.0041†
Non-RP:RP, *N*(%)	78 (63.41):45 (36.59)	64 (69.57):28 (30.43)	14 (45.16):17 (54.84)	0.0147†

Mean ± SD, median (IQR)

^*^Wilcoxon rank-sum test

^†^Chi-square test

The participants consisted of 82 women (66.7%) and 41 men (33.3%), with a mean age of 76.5 ± 6.5 years; 31 out of 123 participants were in the MCI group, and the remainder were in the normal group.

Table 2 shows the demographic characteristics of the participants and a comparison of the objective and subjective masticatory function assessment results between the MCI and normal groups. Regarding the sex distribution ratio, the proportion of females was higher in both the MCI and normal groups, but there was no significant difference in the sex distribution between the two groups (*P* = 0.557). The mean age was significantly higher in the MCI group than in the normal group (*P* = 0.011).

The difference in the KFIA score, reflecting subjective masticatory function, between the normal and MCI groups was not significant, even though there was a significant difference in the MAI, representing objective masticatory function, between the two groups.

Those in the MCI group had significantly fewer remaining teeth and posterior teeth than those in the normal group (*P* = 0.030, < 0.010). Among the objective measures of masticatory function, the bite force in the MCI group was significantly lower than that in the normal group (*P* = 0.048). Regarding the MAI, representing dynamic chewing ability, the MAI was significantly lower in the MCI group than in the normal group (*P* < 0.0001). The Eichner A proportion was higher in the normal group, and the Eichner B + C proportion was significantly higher in the MCI group (*P* = 0.004). The percentage of overall participants wearing RPs was 36.6%, which was more than one-third, and the difference in the percentage of participants wearing RPs between the normal and MCI groups was significant (*P* = 0.015).

### Comparison of masticatory function according to the presence of RPs (Table 3)

**Table 3:** Comparison of masticatory function according to the presence of RPs (*n* = 123)

Dependent variable	Non-RP group (*n* = 78)		*P* value*	RP group (*n* = 45)		*P *value*
	Normal cognition group (*n* = 64)	MCI group (*n* = 14)		Normal cognition group (*n* = 28)	MCI group (*n* = 17)	
Sex M: F	24:40	4:10	0.5281†	8:20	5:12	0.9999†
Age	74.45 ± 5.93, 74 (8.5)	81.71 ± 7 86, 84 (12)	0.0025	77.93 ± 3.84, 78 (5)	77.88 ± 8.07, 78 (14)	0.9628
KMMSE score	27.78 ± 1.58, 28 (2)	19.79 ± 3.42, 20.5 (5)	< .0001	28.11 ± 1.17, 28 (2)	20.29 ± 3.92, 22 (3)	< .0001
KFIA score	3.85 ± 0.88, 3.8 (1.2)	3.69 ± 0.70, 3.5 (1)	0.3329	3.03 ± 1.25	3.04 ± 0.80	0.9999
Remaining teeth	24.45 ± 3.53, 25.5 (5)	23.07 ± 3.45, 23.5 (4)	0.1359	11.71 ± 6.93, 12.5 (11)	12.41 ± 8.05, 12 (10)	0.8795
Posterior teeth	13.44 ± 2.36, 14 (3)	12.21 ± 2.36, 12.5 (2)	0.0736	5 ± 3.94, 4 (7)	5.59 ± 3 68, 6 (6)	0.6317
Bite force	746.46 ± 374.39, 707.25 (542.5)	602.93 ± 406.69, 525.35 (419.4)	0.1334	538.66 ± 392.81, 441.1 (467.6)	469.76 ± 318.87, 391 (487.5)	0.6419
MAI	70.6 ± 7.66, 71.26 (8.04)	56.07 ± 9.8, 58.8 (11.81)	< .0001	68.96 ± 6.79, 70.18 (9.88)	57.05 ± 7.75, 56.42 (11.34)	< .0001
Eichner A: B + C	55:9	9:5	0.0558†	2:26	1:16	0.9999†

Mean ± SD, median (IQR)

^*^Wilcoxon rank-sum test

^†^Fisher's exact test

Table 3 shows a comparison of the results of the subjective and objective masticatory function evaluations according to the presence of RPs. In the non-RP group, there was a significant difference in age and the MAI between the normal and MCI groups (*P* < 0.05). In the RP group, there was a significant difference in the MAI between the MCI and normal groups. That is, the MAI showed a significant difference between the MCI and normal groups regardless of the presence of RPs.

### Association between MCI and masticatory function factors (Table 4)

**Table 4:** Association between MCI and masticatory function factors

Dependent variable	Model 1 (crude)				Model 2 (adjusted)			
	Beta	95% CI		*P* value	Beta	95% CI		*P* value
KFIA score	− 0.27	− 0.69	0.15	0.2042	− 0.03	− 0.45	0.38	0.8723
Remaining teeth	− 3.35	− 6.55	− 0.16	0.0400	− 0.74	− 3.03	1.55	0.5238
Posterior teeth	− 2.29	− 4.26	− 0.32	0.0233	− 0.22	− 1.53	1.10	0.7439
Bite force	− 153.32	− 310.79	4.16	0.0563	− 98.71	− 258.17	60.75	0.2227
MAI	− 13.50	− 16.67	− 10.32	< .0001	− 13.81	− 17.19	− 10.43	< .0001

	Model 1*				Model 2*			
	OR	95% CI		*P* value	OR	95% CI		*P* value
Eichner index B + C (ref. A)	3.42	1.44	8.10	0.0052	3.12	0.77	12.72	0.1124

The independent variable is the MCI group (ref: normal cognition group)

Model 1 is a simple generalized linear model (Model 1* is a simple logistic regression)

Model 2 is a multiple generalized linear model (Model 2* is a multiple logistic regression model) adjusted for sex, age and the presence of RPs

Table 4 shows the results of simple and multiple linear regression analyses of the association of MCI with the factors of the subjective and objective assessments.

The KFIA score and bite force were not significantly different between the MCI and normal groups in the crude model. A significant difference was found in the number of remaining teeth, the number of posterior teeth and the MAI in the MCI group. Additionally, the odds of having incomplete posterior occlusal support were 3.42 times higher in the MCI group than in the normal group according to Model 1* of the simple logistic regression (*P* = 0.005).

Model 2 is a multiple generalized linear model adjusted for age, sex and the presence of RPs, and the results showed that the KFIA score, number of remaining teeth, number of posterior teeth and bite force were not significantly different between the MCI and normal groups. However, a significant difference was shown in the MAI in the MCI group (P < 0.0001). The odds of incomplete posterior occlusal support were 3.12 times higher in the MCI group than in the normal group, with no statistical significance (P = 0.112) in Model 2*.

### Association between MCI and masticatory function factors according to the presence of RPs (Table 5)

**Table 5:** Association between MCI and masticatory function factors according to the presence of RPs

Dependent variable	Multiple generalized linear model*							
	Non-RP group				RP group			
	Beta	95% CI		*P *value*	Beta	95% CI		*P *value*
KFIA score	− 0.10	− 0.65	0.45	0.7203	0.01	− 0.69	0.71	0.9883
Remaining teeth	− 2.14	− 4.40	0.12	0.0633	0.71	− 3.94	5.36	0.7592
Posterior teeth	− 0.98	− 2.51	0.55	0.2077	0.59	− 1.86	3.03	0.6293
Bite force	− 114.87	− 359.87	130.14	0.3532	− 70.99	− 287.91	145.94	0.5124
MAI	− 14.60	− 19.86	− 9.35	< .0001	− 11.85	− 15.87	− 7.83	< .0001

	Multiple logistic regression*							
	Non-RP group				RP group			
	OR	95% CI		*P* value	OR	95% CI		*P* value
Eichner 2 B + C (ref. A)	4.03	0.81	20.18	0.0900	1.36	0.10	18.85	0.8174

The independent variable is the MCI group (ref. the normal cognition group)

^*^Adjusted for sex and age

The influence of the presence of RPs was investigated to determine whether it had any association with cognitive impairment.

Table 5 shows that the KFIA score, number of remaining teeth, number of posterior teeth and bite force had no statistically significant association with MCI regardless of RP use. However, the MAI was significantly lower in the MCI group than in the normal group regardless of the presence of RPs (*P* < 0.0001). Regarding the Eichner index, the likelihood of having incomplete posterior occlusal support was higher in both the non-RP MCI (OR: 4.03, CI 0.81, 20.18) and RP MCI (OR: 1.36, CI 0.10, 18.85) groups, without a significant difference.

## Discussion

Most clinical studies on masticatory function have performed both objective and subjective assessments because while subjective methods show other aspects of mastication, such as adoptive and psychological factors, they have not been used to examine the relationship between MCI and oral function due to the unreliability of data obtained from participants with cognitive decline [7, 12, 16, 23, 33]. However, in this study, both subjective and objective methods were applied because the participants were physically healthy and independent in their daily lives, and chewing ability measured by a subjective masticatory function assessment could have a significantly positive association with cognitive impairment [6]. Therefore, to some extent, it was expected that the results of the subjective and objective masticatory assessments might not necessarily agree completely [34] but that there might be a degree of agreement between them. However, the difference in the KFIA score, reflecting subjective masticatory function, between the normal and MCI groups in this study was not significant (*P* = 0.076), even though there was a significant difference in the MAI, reflecting objective masticatory function. This finding is supported by those previous studies reporting that older people tend to overestimate their physical function without an awareness of latent declines [34]. Additionally, the disagreement rate has been reported to range from 22.4% to 39% [10], and this phenomenon can be explained as ‘anosognosia’, which is a major symptom of MCI originating from the reduced neuronal response in the frontal and parietal cortical midline structures [24, 35].

Among structural factors, such as the number of remaining teeth, the number of posterior teeth, the condition of posterior support and the presence of restorations, the number of teeth has been focused on as a factor related to general cognitive function [8, 9, 13, 21]. However, in the present study, there was a significant difference in the number of teeth and in the number of posterior teeth, but there was no significant association between the number of remaining teeth or posterior teeth alone and cognitive impairment regardless of age, sex, and the presence of RPs (Table 4). Most of the participants in previous studies reporting a significant association between the number of teeth and cognitive ability were edentulous or had fewer than 10 teeth [23, 36]. However, in this study, the mean number of teeth in the normal and MCI groups was 22.50 (20.58 ± 7.5) and 19.00 (17.23 ± 8.30), respectively, which can be considered functionally acceptable based on the shortened dental arch concept [37]. Based on these results, it can be estimated that the number of teeth is not significantly associated with cognitive ability under conditions of approximately 20 teeth, which can be supported by previous studies reporting no significant association between the number of teeth and cognitive ability for populations with a mean of 20 teeth [16, 33].

On the other hand, elderly people are more likely to lose teeth, and this tendency becomes more apparent as aging progresses; therefore, it is difficult to determine the validity of assessing the number of remaining teeth in investigating associations with cognitive function. Ikebe et al. reported that the number of teeth was significantly related to the cognitive score in a group of participants in their 70 s but not in a group of participants in their 80 s and that the occlusal force had a statistically significant association with cognitive function in both those in their 70 s and in their 80 s [16]. On the basis of these findings, it can be speculated that most of the elderly individuals in their 80 s had experienced tooth loss, resulting in the lack of a significant association with cognitive function.

In this study, only the MAI among the objective variables appeared to have a significant positive correlation with MCI regardless of age, sex, and the presence of RPs (*P* < 0.0001). Although there were methodological differences, this result could be supported by previous studies reporting that masticatory efficiency, that is, whether a subject can chew without difficulty, is a key factor in the executive function of cognitive ability [6, 7, 10, 16, 23, 36].

The posterior occlusal contact condition was assessed using the Eichner index to determine the degree of dental morbidity. Several studies have emphasized the importance of posterior occlusal contact in masticatory performance and cognitive function [38, 39]. However, even though the simple ratio comparison between Eichner B + C and Eichner A showed a significant difference between the MCI and normal groups (Table 2, *P* = 0.004) and the odds for Eichner B + C compared to Eichner A was 3.12 times higher in the MCI group in the multiple generalized logistic regression, a significant association with MCI could not be found in this study.

Further studies of sufficient numbers of participants with various occlusal posterior support conditions and without RPs using more specific methods, such as examination of the number of functional tooth units (FTUs) or the occlusal contact area, are needed for a more accurate investigation of the association between posterior occlusion and MCI.

In contrast to a previous study reporting that bite force is related to cognitive function [15, 16, 40], no significant association between bite force and cognitive function was found in this study. Chewing is a series of processes in which the teeth, muscles, and neuromuscular system continuously cooperate for a certain period of time; therefore, the bite force, which represents the force temporarily exerted at a specific moment, unlike the MAI, has a limitation as an objective masticatory functional factor for predicting masticatory performance [41].

There are some limitations to this study. The KMMSE was used for general screening for cognitive impairment instead of an extensive neuropsychological assessment tool. The MMSE, originally developed for general screening in a hospital, has been reported to have a ‘false-negative’ response rate as high as 19.7–30% [26]. For further investigation of cognitive function, the accuracy of testing could be improved by incorporating a more extensive neuropsychological testing tool for the aspects of cognitive function.

The power of this analysis was greater than 0.8, and the significance level was sufficiently high; thus, our results can be considered reliable. However, for more precise verification, a sufficient number of participants should be included in the MCI and normal groups with a uniform sex distribution. In addition, care must be taken to select participants with various oral conditions, such as various degrees of posterior occlusal support and types of prostheses, to avoid possible bias in the results.

## Conclusion

Based on data from participants with approximately 20 remaining teeth and no difficulty chewing, the MAI, among other masticatory factors, was significantly associated with MCI in elderly patients.

## Supplementary Information

Below is the link to the electronic supplementary material.

Supplementary file1 (XLSX 22 KB)
